# Identifying Relevant Covariates in RNA-seq Analysis by Pseudo-Variable Augmentation

**DOI:** 10.1007/s13253-024-00665-3

**Published:** 2024-11-02

**Authors:** Yet Nguyen, Dan Nettleton

**Affiliations:** 1 Department of Mathematics and Statistics, Old Dominion University, Norfolk, VA 23529 USA; 2 Department of Statistics, Iowa State University, Ames, IA 50011 USA

**Keywords:** False selection rate, False discovery rate, Variable selection, Differential expression analysis

## Abstract

RNA-sequencing (RNA-seq) technology allows for the identification of differentially expressed genes, which are genes whose mean transcript abundance levels vary across conditions. In practice, RNA-seq datasets often include covariates that are of primary interest in addition to a set of covariates that are subject to selection. Some of these covariates may be relevant to gene expression levels, while others may be irrelevant. Ignoring relevant covariates or attempting to adjust for the effect of irrelevant covariates can compromise the identification of differentially expressed genes. To address this issue, we propose a variable selection method that uses pseudo-variables to control the expected proportion of selected covariates that are irrelevant. Our method accurately selects relevant covariates while keeping the false selection rate below a specified level. We demonstrate that our method outperforms existing methods for detecting differentially expressed genes when working with available covariates. Our method is implemented in FSRAnalysisBS function of the R package csrnaseq, which is available at www.github.com/ntyet/csrnaseq. The analysis and simulation are available at www.github.com/ntyet/csrnaseq/tree/main/analysis.

**Supplementary Information:**

The online version contains supplementary material available at 10.1007/s13253-024-00665-3.

## Introduction

In experiments or studies that measure RNA transcript abundance levels via RNA-seq technology, a common goal is to understand the association between the RNA levels of each gene (i.e., gene expression) and one or more explanatory variables using a regression model. Some explanatory variables must be included in regression models for gene expression because of scientific interest (e.g., a treatment factor) or because the design of the study or experiment dictates their inclusion (e.g., the blocking factor in a randomized complete block design). We use *primary variables* to refer to these explanatory variables that are not subject to variable selection in regression models for gene expression analysis. In many cases, other explanatory variables, which we refer to as *covariates*, are measured but subject to variable selection. *Relevant covariates* hold important information about the experimental/observational units or about factors associated with the complex process of measuring RNA levels using RNA-seq technology. In contrast to relevant covariates, *irrelevant covariates* have no important associations with gene expression levels when primary variables and relevant covariates are accounted for.

Distinguishing the relevant covariates from the irrelevant covariates is important for differential expression analysis. A gene is defined as differentially expressed (DE) with respect to a primary variable if the gene’s expression is associated with the primary variable after controlling for the effects of other primary variables and relevant covariates. A gene not differentially expressed is said to be equivalently expressed (EE). DE genes can be detected by testing the significance of partial regression coefficients in regression models that include primary variables and relevant covariates. Detecting DE genes is challenging, in part, because the status of each covariate as relevant or irrelevant is unknown. Ignoring relevant covariates or attempting to adjust for the effect of irrelevant covariates can compromise the identification of DE genes. The former can lead to biased estimators of regression coefficients and inflated estimates of error variance, while the latter can lead to increases in the variance of parameter estimators, which diminishes the power of tests for differential expression. Thus, selecting the subset of relevant covariates has a critical role in differential expression analysis.

There exist numerous methods for conducting differential expression analysis on RNA-seq data, including QuasiSeq (Lund et al. 2012; Lun et al. 2016), edgeR (McCarthy et al. 2012), DESeq2 (Love et al. 2014), and voom (Law et al. 2014), among others, as extensively reviewed by Costa-Silva et al. (2017). However, these methods do not offer specific recommendations or strategies for handling variable selection in RNA-seq differential expression analysis. Nguyen et al. (2015) addressed the covariate selection problem in the context of RNA-seq differential expression analysis by proposing a backward selection strategy to choose the most relevant covariates. Nguyen et al. (2015)’s method works well when the primary variables are uncorrelated or weakly correlated with one or more covariates. However, if one or more covariates are strongly associated with the primary variables, their method fails to detect the truly relevant covariates and may result in a high number of false positives, resulting in the failure to control false discovery rate (FDR).

In this paper, we introduce a new covariate selection strategy in RNA-seq analysis that can overcome the limitation of the aforementioned method. Our method is based on a variable selection approach originally designed to control the false selection rate (FSR) in linear regression for one response variable (Wu et al. 2007). Wu et al. (2007)’s method involves augmenting the set of available covariates with pseudo-variables that, by construction, are known to be irrelevant variables that should not be included in the model for the single response variable. By studying a selection method’s propensity to include pseudo-variables among the selected set of covariates, it is often possible to tune selection to control FSR below a specified threshold. Wu et al. (2007) use forward selection with the $p$-value associated with each covariate as the measure of covariate relevance. We extend the method of Wu et al. (2007) to multiple response variables so that the method can be used in differential expression analysis, where there is one response variable for each of thousands of genes. Instead of having only one $p$-value, a covariate in the case of multiple response variables has a vector of $p$-values, with one $p$-value for each response variable. To account for the whole vector of $p$-values associated with a covariate, we propose a simple covariate relevance measure that can be described, informally, as the ratio of the number of small $p$-values to the number of large $p$-values. If a covariate’s relevance measure is close to 1, it indicates that the covariate is irrelevant. Conversely, if a covariate’s relevance measure is substantially greater than 1, it signifies high relevance of the covariate. For a given covariate, the greater the ratio, the more relevant the covariate is judged to be.

We illustrate the performance of our method using a backward selection procedure and the differential expression analysis method voom-limma (Law et al. 2014). If covariates are uncorrelated or weakly correlated with the primary variables, the performance of our method is similar to that of Nguyen et al. (2015). On the other hand, if there are covariates strongly correlated with the primary variables, our method outperforms the method of Nguyen et al. (2015). We also compare the differential expression analysis produced by our method with alternatives for handling covariates in differential expression analysis, such as the method of Nguyen et al. (2015) and methods that forgo selection by either including or excluding all covariates. As a result of accounting for truly relevant covariates, our approach outperforms others in terms of power, FDR control, and ability to distinguish the true and false signals with respect to the primary variables.

The paper is organized as follows. In Sect. 2, we first give general preliminaries about RNA-seq data, available covariates, and the voom method; then, we explain the proposed covariate selection method using pseudo-variables. In Sect. 3, we apply our method to analyze an RNA-seq dataset with many covariates from a residual feed intake (RFI) experiment conducted to find genes differentially expressed between two RFI lines. In Sect. 4, we conduct a data-driven simulation study to investigate the performance of our method relative to competing methods in the ability to select truly relevant covariates and to identify DE genes. Section 4 is presented following the ADEMP (Aims, Data-generating mechanisms, Estimand, Methods, Performance measures) scheme proposed by Morris et al. (2019). Finally, Sect. 5 is devoted to a conclusion and discussion.

## Method

### Notations and Preliminaries

Consider the analysis of $G$ genes using RNA-seq read count data from $n$ subjects. Let ${\boldsymbol{x}}_{i\cdot } = ({\boldsymbol{x}}'_{i1}, \dots , {\boldsymbol{x}}'_{ij}, \dots , {\boldsymbol{x}}'_{ik})'$ be the vector of known $k$ explanatory variable values for subject $i = 1, \dots , n$, and let ${\boldsymbol{x}}_{\cdot j}$ be a matrix of $n$-dimensional column vectors corresponding to the $j$-th variable for $j = 1, \dots , k$. The matrix ${\boldsymbol{x}}_{\cdot j}$ for a continuous variable $j$ has exactly one column while ${\boldsymbol{x}}_{\cdot j}$ corresponding to a categorical variable $j$ consists of columns corresponding to the variable’s level indicators with one less indicator than the number of levels of the categorical variable. Without loss of generality, we assume $\{{\boldsymbol{x}}_{\cdot 1}, \dots , {\boldsymbol{x}}_{\cdot \ell }\}$ to be the primary variables, and $\{ {\boldsymbol{x}}_{\cdot \ell +1}, \dots , {\boldsymbol{x}}_{\cdot k}\}$ to be the covariates subject to variable selection.

Let $c_{gi}$ be the read count from gene $g$ and sample $i$, and let $R_i = \sum _{g = 1}^G c_{gi}$, which is known as the library size of the RNA-seq sample for subject $i$. The log-counts per million (Law et al. 2014) is defined as

1 $$\begin{aligned} y_{gi} = \log _2\left( \frac{c_{gi} +0.5}{R_i +1} \times 10^6\right) . \end{aligned}$$

We set $R_i$ to be the $75^{th}$ percentile (or upper-quartile) of RNA-seq sample read counts from subject $i$ according to the recommendation of Bullard et al. (2010). With this choice of normalization offsets, the $y_{gi}$ values are no longer “counts per million mapped reads” on the log scale, but this interpretation is irrelevant for our RNA-seq covariate selection and differential expression analysis. Henceforth, we use $\boldsymbol{c}_g = (c_{g1}, \dots , c_{gn})'$ and $\boldsymbol{y}_g = (y_{g1}, \dots , y_{gn})'$ to denote the vector of count values and the vector of log-count values for gene $g$.

### voom Procedure

In this paper, we use the voom method because of its good false discovery rate control, power, and computational speed. voom is based on linear model analysis that incorporates the mean-variance relationship of the log-counts by introducing a precision weight for each observation according to the following algorithm.

1. Let ${\mathcal {S}}$ represent a subset of $\{1, \dots , k\}$ that contains $\{1, \dots , \ell \}$, the indices of the $\ell$ primary variables. For each gene *g*, assume a linear model
  2 $$\begin{aligned} &  y_{gi} =\beta _{g0|\mathcal {S}} + \sum _{j \in \mathcal {S}}{\boldsymbol{x}}_{ij}'\boldsymbol{\beta }_{gj|\mathcal {S}} + \varepsilon _{gi|\mathcal {S}},\quad \varepsilon _{gi|\mathcal {S}} \sim {{{\mathcal {N}}}}(0, \sigma ^2_{g|\mathcal {S}}), \quad g = 1, \nonumber \\  &  \dots , G;\; i = 1, \dots ,n \end{aligned}$$
  or equivalently, in vector form,
  3 $$\begin{aligned} \boldsymbol{y}_g= {\boldsymbol{X}}_{\mathcal {S}}\boldsymbol{\beta }_{g|\mathcal {S}} + \boldsymbol{\varepsilon }_{g|\mathcal {S}}. \end{aligned}$$
  where ${\boldsymbol{X}}_{\mathcal {S}}$ is the design matrix consisting of an intercept column $\boldsymbol{1}$ and all columns in ${\boldsymbol{x}}_{\cdot j}$ for all $j \in \mathcal {S}$; $\boldsymbol{\beta }_{g|\mathcal {S}}$ is the vector of regression coefficients consisting of $\beta _{g0|\mathcal {S}}$ and all $\boldsymbol{\beta }_{gj|\mathcal {S}}$ for $j \in \mathcal {S}$.
2. Let $\widehat{\boldsymbol{\beta }}_{g|\mathcal {S}} = ({\boldsymbol{X}}_{\mathcal {S}}'{\boldsymbol{X}}_{\mathcal {S}})^{-1}{\boldsymbol{X}}_{\mathcal {S}}'\boldsymbol{y}_g$ and $s_{g|\mathcal {S}} = \sqrt{\frac{\left( \boldsymbol{y}_g - {\boldsymbol{X}}_{\mathcal {S}}\widehat{\boldsymbol{\beta }}_{g|\mathcal {S}}\right) '\left( \boldsymbol{y}_g - {\boldsymbol{X}}_{\mathcal {S}}\widehat{\boldsymbol{\beta }}_{g|\mathcal {S}}\right) }{n - \mathrm{ rank }({\boldsymbol{X}}_{\mathcal {S}}) }}$ be the ML and REML estimates of $\boldsymbol{\beta }_{g|\mathcal {S}}$ and $\sigma _{g|\mathcal {S}}$, respectively. Let $\widehat{\boldsymbol{y}}_g = {\boldsymbol{X}}_{\mathcal {S}}\widehat{\boldsymbol{\beta }}_{g|\mathcal {S}}$.
3. Let ${\tilde{c}}_g = \frac{1}{n}\sum _{i = 1}^ny_{gi} +\frac{1}{n}\log _2 \left( \prod _{i = 1}^n(R_i+1)\right) - \log _2(10^6)$ be the mean log-count value for each gene *g*.
4. Let $\text{ lo }(\cdot )$ be the predictor obtained by fitting a LOWESS regression (Cleveland 1979) of $s_{g|\mathcal {S}}^{1/2}$ on ${\tilde{c}}_g$. The precision weight for $y_{gi}$ is calculated by
  $$\begin{aligned} w_{gi} = \left[ \text{ lo }\left( {\widehat{y}}_{gi} + \log _2(R_i+1) - \log _2(10^6) \right) \right] ^{-4}. \end{aligned}$$

The normalized log-counts and their associated precision weights then enter the limma pipeline for downstream analysis, including shrinkage estimation of error variances and calculation of moderated $t$-statistics or moderated $F$-statistics for partial regression coefficients (Smyth 2004; Ritchie et al. 2015). These statistics are then compared to a central $t$ or $F$ distribution to obtain $p$-values, which are converted to $q$-values by the method of Storey (2002) in combination with the histogram-based method of Nettleton et al. (2006) for an estimation of the number of true null hypotheses among all null hypotheses tested. Finally, a gene is declared as differentially expressed (DE) if its $q$-value is smaller than or equal to a nominal false discovery rate level, typically 5%, otherwise, the gene is declared as equivalently expressed (EE). To simplify the notation, in what follows, we drop the subscripts ${\mathcal {S}}$ and $|{\mathcal {S}}$, while keeping in mind that the inference about any particular covariate is conducted by conditioning on the other explanatory variables included in the model.

### Measure of Covariate Relevance

For a given $j \in {{\mathcal {S}}} {\setminus } \{1, \dots , \ell \}$, let $\boldsymbol{p}_{j}$ be the vector of $G$ $p$-values obtained by testing $H_{0gj}: \boldsymbol{\beta }_{gj} =\textbf{0}$ for each gene $g= 1, \dots , G$ via limma. We consider covariate $j$ irrelevant if

4 $$\begin{aligned} H_{0gj}: \boldsymbol{\beta }_{gj} = \textbf{0} \hbox { is true for all } g = 1, \dots , G. \end{aligned}$$

If (4) is met, each element of $\boldsymbol{p}_{j}$ follows a uniform distribution on $(0,1)$ whenever the test used to generate these elements has size equal to the significance level for all significance levels in $(0,1)$. When the test employed to generate the elements of $\boldsymbol{p}_{j}$ remains unbiased across all significance levels within $(0,1)$, an element of $\boldsymbol{p}_{j}$ associated with a false null hypothesis will exhibit a distribution that is stochastically smaller than the uniform distribution on $(0,1)$ and a density that decreases over the interval $(0,1)$. Therefore, an empirical distribution of $\boldsymbol{p}_{j}$ close to uniform(0, 1) or stochastically larger than uniform(0, 1) implies little relevance while an empirical distribution with clear excess of small $p$-values relative to uniform(0, 1) implies relevance of covariate $j$. This motivates us to propose an intuitive covariate relevance measure which is formally stated in the following definition.

#### Definition 1

With $1\!\!1$ representing an indicator function, a relevance measure for covariate *j* is defined as

5 $$\begin{aligned} r(\boldsymbol{p}_{j}) = \frac{\sum _{g = 1}^{G} 1\!\!1(p_{gj} \le 0.05)}{\max \{\sum _{g = 1}^{G} 1\!\!1(p_{gj} \ge 0.75)/5, 1\}}. \end{aligned}$$

**Fig. 1:**
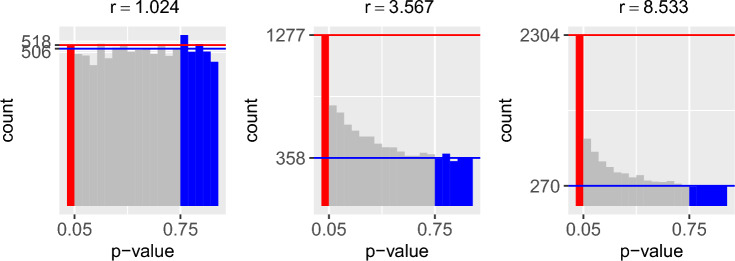
An example is shown to illustrate the relevance level of a covariate measured by the *r* function. Each subplot represents an instance of a covariate with different relevance levels, with values of $r = 1.024$ representing an irrelevant covariate, $r = 3.567$ representing a relevant covariate, and $r = 8.533$ representing a highly relevant covariate

In (5), if $r(\boldsymbol{p}_{j}) \le 1$, the number of large $p$-values exceeds the number of small $p$-values, which suggests covariate $j$ is irrelevant or less important. On the other hand, if $r(\boldsymbol{p}_{j}) \gg 1$, the number of small $p$-values is much greater than the number of large $p$-values, which suggests covariate $j$ is relevant or highly important (see, Fig. 1). In other words, the relevance measure $r(\boldsymbol{p}_{j})$ quantifies the importance of covariate $j$ in explaining gene expression levels, with higher values indicating greater relevance and lower values indicating less relevance. We combine this measurement with the FSR variable selection method (Wu et al. 2007), which we will review in the next subsection.

### False Selection Rate Variable Selection Method

Commonly used approaches to select an important subset of variables in general multiple regression problems include all subsets, backward selection, forward selection, and step-wise selection using some measurement of variable importance. In addition, there are other variable selection methods using regularization, see, e.g., LASSO, SCAD, Least Angle Regression, etc. These methods are collectively reviewed by Heinze et al. (2018).

A good variable selection procedure will, on average, include a high percentage of the relevant covariates and a low percentage of the irrelevant ones. The false selection rate (FSR) method proposed by Wu et al. (2007) uses a novel criterion aiming to control the average proportion of selected variables that are irrelevant. Their method is based on a simple idea that the tendency of a variable selection method to overfit or underfit can be revealed by the use of pseudo-variables. For completeness, we review in the next subsection the details of their method, adapted for gene expression data. While the original work in Wu et al. (2007) was demonstrated for a forward selection strategy, in this paper, we describe the method for a backward selection strategy, for the purpose of illustrating the FSR method in RNA-seq analysis, and for comparison with the backward selection strategy in Nguyen et al. (2015). The idea of using pseudo-variables in the FSR method is applicable to other variable selection algorithms as well.

#### FSR for Backward Selection

For simplicity, let ${\boldsymbol{C}} = [{\boldsymbol{c}}_1, \dots , {\boldsymbol{c}}_G]'$ be the matrix of counts RNA-seq data with one row for each gene and one column for each sample. Let ${\boldsymbol{X}} = [{\boldsymbol{X}}_1, {\boldsymbol{X}}_2]$, where ${\boldsymbol{X}}_1 = [{\boldsymbol{x}}_{\cdot 1}, \dots , {\boldsymbol{x}}_{\cdot \ell }]$, and ${\boldsymbol{X}}_2 = [{\boldsymbol{x}}_{\cdot \ell + 1}, \dots , {\boldsymbol{x}}_{\cdot k}]$. The backward selection procedure starts by fitting the procedure described in Sects. 2.1 and 2.2 to ${\boldsymbol{C}}$ and ${\boldsymbol{X}}$. Using the relevance measure $r$ described in Sect. 2.3, the least relevant variable in ${\boldsymbol{X}}_2$ is removed, and its $r$ value is recorded. The resulting reduced model is fit again using voom. The process of removing variables and refitting continues until the last variable in ${\boldsymbol{X}}_2$ is removed. This backward selection procedure produces a sequence of increasingly smaller subsets of variables, starting with all variables in ${\boldsymbol{X}}$ and progressing, one removed covariate (in ${\boldsymbol{X}}_2$) at a time, down to ${\boldsymbol{X}}_1$. For a given cut-off threshold $\lambda$, let $BS(({\boldsymbol{C}}; {\boldsymbol{X}}_1; {\boldsymbol{X}}_2), \lambda )$ denote the subset of ${\boldsymbol{X}}_2$ selected by this backward selection, i.e., the largest subset of ${\boldsymbol{X}}_2$ for which each variable has $r$-value at least $\lambda$. Define $S(\lambda ) = \hbox {Card}\{BS(({\boldsymbol{C}};{\boldsymbol{X}}_1; {\boldsymbol{X}}_2),\lambda )\}$. Then $S(\lambda ) = R(\lambda ) + I(\lambda )$, where $R(\lambda )$ and $I(\lambda )$ denote the number of selected relevant and irrelevant covariates, respectively.

To control the proportion of selected covariates that are irrelevant, Wu et al. (2007) defined two FSR functions as

6 $$\begin{aligned} \alpha _{RE}(\lambda ) = \frac{E(I(\lambda ))}{E(1+S(\lambda ))} = \frac{E(I(\lambda ))}{E(1+R(\lambda )+I(\lambda )))}, \end{aligned}$$

and

7 $$\begin{aligned} \alpha _{ER}(\lambda ) = E\left( \frac{I(\lambda )}{1+S(\lambda )}\right) = E\left( \frac{I(\lambda )}{1+R(\lambda )+I(\lambda )}\right) \end{aligned}$$

where *RE* means *Ratio of Expectation* and *ER* means *Expectation of Ratio*. The FSR variable selection method aims to determine the critical value $\lambda _*$ such that $\alpha _\cdot (\lambda _*) \le \alpha _0$ for some pre-specified FSR threshold $\alpha _0$, say $\alpha _0 = 0.05$; where $\alpha _\cdot$ denotes either $\alpha _{RE}$ or $\alpha _{ER}$. Formally, $\lambda _*$ is defined as

$$\begin{aligned} \lambda _* = \inf \{\lambda : \alpha _\cdot (\lambda ) \le \alpha _0\}, \end{aligned}$$

so that the largest model consistent with controlling false selection to include as many important covariates as possible. Because $\alpha _{\cdot }(\cdot )$ is unknown, $\lambda _*$ cannot be determined directly. Wu et al. (2007) showed that it can be estimated approximately using Monte-Carlo generated pseudo-variables as follows.

For some integer number $B$ and each $b = 1, \dots , B$, suppose that ${\boldsymbol{Z}}_b$ is a set of $k_P$ pseudo-variables that are randomly generated to be independent of the response variables ${\boldsymbol{C}}$. The backward selection procedure described previously is applied to ${\boldsymbol{C}}$ and $[{\boldsymbol{X}}_1, {\boldsymbol{X}}_2, {\boldsymbol{Z}}_b]$ where now $[{\boldsymbol{X}}_2, {\boldsymbol{Z}}_b]$ is the set of covariates that are subject to variable selection. Let $S_{P, b}(\lambda )$ be the total number of selected covariates. Let $R_{P,b}(\lambda )$ and $I_{P,b}(\lambda )$ be the number of selected relevant and irrelevant covariates, respectively. Let $I^*_{P,b}(\lambda )$ be the number of selected pseudo-variables. Then $S_{P,b}(\lambda ) = R_{P,b}(\lambda ) + I_{P,b}(\lambda ) + I^*_{P,b}(\lambda )$. To estimate $\alpha _{\cdot }(\lambda )$, Wu et al. (2007) assumed further that

1. $E(I(\lambda )) = E(I_{P,b}(\lambda )) = k_I E(I^*_{P,b}(\lambda ))/k_P$, where $k_I$ is the unknown number of irrelevant covariates.
2. $$E(R_{P,b}(\lambda )) = E(R(\lambda ))$$.

Assumption (A1) states that on average real irrelevant covariates and pseudo-variables have the same probability of being selected. Assumption (A2) states that on average the truly relevant covariates have the same probability of being selected whether or not pseudo-variables are present. These assumptions are considered as guiding principles for generating pseudo-variables, estimating FSR and estimating $\lambda _*$, rather than as crucial mathematical conditions justifying the methods. In next subsections, we describe how FSR method works using each of the FSR functions.

#### FSR Method Based on Estimating $\alpha _{RE}(\lambda )$

First, define

$$\begin{aligned} \alpha _{RE,P}(\lambda ) = \frac{E(I^*_{P,b}(\lambda ))}{E(1+S_{P,b}(\lambda ))}. \end{aligned}$$

Then using assumptions (A1) and (A2), we have

8 $$\begin{aligned} \alpha _{RE,P}(\lambda )&= \frac{E(I^*_{P,b}(\lambda ))}{1+E(R_{P,b}(\lambda )) +E(I_{P, b}(\lambda )) +E(I^*_{P,b}(\lambda ))}\nonumber \\&=\frac{k_P E(I(\lambda ))/k_I}{1+E(R(\lambda )) +E(I(\lambda )) +k_P E(I(\lambda ))/k_I}\nonumber \\&=\frac{k_P E(I(\lambda ))/k_I}{E(1+R(\lambda ) +I(\lambda )) +k_P E(I(\lambda ))/k_I}\nonumber \\&=\frac{k_P \frac{E(I(\lambda ))}{E(1+R(\lambda ) +I(\lambda ))}/k_I}{\frac{E(1+R(\lambda ) +I(\lambda )) +k_P E(I(\lambda ))/k_I}{E(1+R(\lambda ) +I(\lambda ))}}\nonumber \\&= \frac{k_P \alpha _{RE}(\lambda )/k_I}{1+k_P\alpha _{RE}(\lambda )/k_I} = \frac{k_P\alpha _{RE}(\lambda )}{k_P\alpha _{RE}(\lambda ) + k_I}. \end{aligned}$$

Moreover, $\alpha _{RE,P}(\lambda )$ is estimated by

9 $$\begin{aligned} \widehat{\alpha }_{RE,P}(\lambda ) = \frac{{\bar{I}}^*_{P}(\lambda )}{1+{\bar{S}}_P(\lambda )}, \end{aligned}$$

where

$$\begin{aligned} {\bar{I}}^*_{P}(\lambda ) =B^{-1}\sum _{b=1}^B I^*_{P,b}(\lambda ), {\bar{S}}_P(\lambda ) = B^{-1}\sum _{b=1}^B S_{P,b}(\lambda ). \end{aligned}$$

Using (8) and (9), $\alpha _{RE}(\lambda )$ is estimated as the solution to the following equation

$$\begin{aligned} \widehat{\alpha }_{RE,P}(\lambda ) = \frac{k_P\alpha _{RE}(\lambda )}{k_P\alpha _{RE}(\lambda ) + k_I}, \end{aligned}$$

whose the right-hand side is a monotone increasing function of $\alpha _{RE}(\lambda )$. Therefore, if $k_I$ is known, for a given FSR level $\alpha _0$, an estimate $\widehat{\lambda }_*$ of $\lambda _*$ can be obtained as

10 $$\begin{aligned} \widehat{\lambda }_* =\inf \left\{ \lambda : \widehat{\alpha }_{RE,P} (\lambda ) \le c:=\frac{k_p\alpha _0}{k_p\alpha _0 + k_I}\right\} . \end{aligned}$$

In general, $k_I$ is unknown. Wu (2004) proposed an iterative algorithm to estimate $k_I$ which in turn is used to estimate $\lambda _*$ via (10). The algorithm adapted for backward selection and RNA-seq analysis is summarized as in Sect. 3 of the accompany Supplementary Materials.

#### FSR Method Based on Estimating $\alpha _{ER}(\lambda )$

Similar to what described in Sect. 2.4.2, Wu et al. (2007) defined

11 $$\begin{aligned} \alpha _{ER,P}(\lambda )&= \frac{E(I_{P,b}^*(\lambda ))}{1+S(\lambda )} = \frac{k_P E(I(\lambda ))/k_I}{1+S(\lambda )}\nonumber \\&\approx k_P E\left( \frac{I(\lambda )}{1+S(\lambda )} \right) /k_I \approx k_P \alpha _{ER}(\lambda )/k_I. \end{aligned}$$

On the other hand, $\alpha _{ER,P}(\lambda )$ is estimated by

12 $$\begin{aligned} \widehat{\alpha }_{ER,P}(\lambda ) = \frac{{\bar{I}}^*_{P}(\lambda )}{1+S(\lambda )}. \end{aligned}$$

Combining (11) and (12), $\alpha _{ER}(\lambda )$ is estimated as the solution to the following equation

$$\begin{aligned} \widehat{\alpha }_{ER,P}(\lambda ) = \frac{k_P}{k_I}\alpha _{ER}(\lambda ). \end{aligned}$$

Therefore, if $k_I$ is known, for a given FSR level $\alpha _0$, an estimate of $\lambda _*$ is obtained by

$$\begin{aligned} \widehat{\lambda }_* = \inf \left\{ \lambda : \widehat{\alpha }_{ER,P}(\lambda ) \le c:=\frac{k_P}{k_I}\alpha _0\right\} . \end{aligned}$$

Then, an FSR algorithm for estimation of $k_I$ and $\lambda _*$ using $\alpha _{ER}$ is described as in Sect. 4 of the accompany Supplementary Materials.

#### Pseudo-Variable Generation

In Sect. 2.4.1, we mentioned that pseudo-variables will be generated according to conditions (A1) and (A2), so that the average inclusion probabilities of relevant and irrelevant covariates are approximately equal for data $({\boldsymbol{C}}; {\boldsymbol{X}}_1; {\boldsymbol{X}}_2)$ and $({\boldsymbol{C}}; {\boldsymbol{X}}_1;{\boldsymbol{X}}_2,{\boldsymbol{Z}})$, where ${\boldsymbol{Z}}$ is a set of $k_P$ randomly generated pseudo-variables. Based on these principles, Wu et al. (2007) proposed four different methods to generate pseudo-variables.

The first method involves generating entries of the $n\times k_P$ matrix ${\boldsymbol{Z}}$ independently and identically distributed from $N(0,1)$. The second method involves obtaining the $n$ rows of ${\boldsymbol{Z}}$ by randomly selecting $k_P$ columns of ${\boldsymbol{X}}$ and randomly permuting the $n$ rows of the resulting $n\times k_P$ matrix. In both methods, the pseudo-variables are stochastically uncorrelated with the original explanatory variables, while in the second method, the pseudo-variables have the same distribution as a subset of the explanatory variables.

The third and fourth methods are variants of the first two methods, in which ${\boldsymbol{Z}}$ is replaced by $(\boldsymbol{I} - \boldsymbol{H}){\boldsymbol{Z}}$, where $\boldsymbol{H} = {\boldsymbol{X}}({\boldsymbol{X}}'{\boldsymbol{X}})^{-1}{\boldsymbol{X}}'$. The variants are such that the pseudo-variables have sample means and sample correlations with the explanatory variables identically equal to 0. Note that the variants are only possible when the rank of the linear space generated by the explanatory variables (including the intercept, the primary variables, and the covariates subject to variable selection) is smaller than the number of RNA-seq samples, which is the case when a backward selection strategy can be applied. If the number of explanatory variables is large, a forward selection strategy can be implemented together with the FSR method. However, in that case, only the first two pseudo-variable generating methods can be used.

We refer to the first two pseudo-variable generating methods as WN (white noise $N(0,1)$) and RX (permuting rows of ${\boldsymbol{X}}$), and to their variant versions as OWN and ORX, respectively. We also denote the eight variants of the FSR method, which are obtained by combining the two methods of estimating FSR ($\alpha _{RE}$ and $\alpha _{ER}$) with the four pseudo-variable generating mechanisms (WN, RX, OWN, and ORX), as follows: WN$\_$RE, WN$\_$ER, RX$\_$RE, RX$\_$ER, OWN$\_$RE, OWN$\_$ER, ORX$\_$RE, ORX$\_$ER.

Our method, implemented in the FSRAnalysisBS function of the R package csrnaseq available at www.github.com/ntyet/csrnaseq, is summarized by the following steps:

1. Perform backward selection on the original RNA-seq dataset with the available covariates.
2. Generate $B$ sets of $k_P$ pseudo-variables to estimate FSR based on algorithms in Sects. 2.4.2 and 2.4.3.
3. Estimate the final critical value ${\hat{\lambda }}_*$ which then is used to identify the selected covariates in the backward selection algorithm on the original dataset.
4. Utilize the final selected covariates to conduct differential expression analysis.

## Real Data Analysis

Now we apply the FSR variable selection method to the RFI RNA-seq dataset. This dataset has been used and described in Nguyen et al. (2015) and Liu et al. (2016). The dataset is available at https://www.ebi.ac.uk/arrayexpress/ under accession number E-MTAB-4179. For completeness, we summarize the RFI dataset as follows.

Residual feed intake (RFI) is an important quantitative trait measuring feed efficiency. It is calculated as the difference between the observed feed intake and the expected feed intake considering the animal’s growth. A pig with low RFI tends to make use of food more efficiently than normal pigs, while a pig with high RFI uses food less efficiently than normal pigs. Researchers are interested in finding genes whose expression levels differ between two *lines* of pigs, known as the high RFI line and the low RFI line, created via divergent selection. The analysis is complex because of heterogeneity among pigs, among blood samples collected from pigs, and among the processed and measured RNA samples derived from the blood samples. The heterogeneity is shown in associated covariates that are measured along with RNA-seq samples such as each animal’s diet (*Diet*: a categorical factor with two levels), RFI value of each animal (*RFI*: a continuous variable), quality of RNA-seq samples (*Concb*, *RINb*, *Conca*, *RINa*: continuous covariates of RNA *Conc*entration and *R*NA *I*ntegrity *N*umber *b*efore and *a*fter globin depletion), characteristics of blood samples (*Baso, Eosi, Lymp, Mono, Neut*: continuous covariates of the concentration of *baso*phil, *eosi*nophil, *lymp*hocyte, *mono*cyte, *neut*rophil cells in the blood sample drawn from each pig) and the process of measuring RNA-seq samples, captured by experimental design factors (*Block*: a categorical factor with four levels corresponding to the four blocks used to organize sample collection and processing, *Order*: a categorical factor with eight levels indicating the random order samples were processed within each block). For more detailed information on the variables of the dataset, please refer to Sects. 1 and 2 of the accompany Supplementary Materials.

In summary, the RFI RNA-seq dataset consists of 12280 genes that each have average read counts of at least 8 and have no more than 27 zero counts out of 31 pigs. The primary variable is *Line* with two levels, low RFI and high RFI. *Line* is the only variable of primary scientific interest that is not subject to variable selection. The available covariates subject to variable selection are *Diet* (2 levels), *Order* (8 levels), *Block* (4 levels), *Concb, RINb, Conca, RINa, Baso, Eosi, Lymp, Mono, Neut*, and *RFI* (continuous covariates). Then, we have two sets of variables:

- ${\boldsymbol{X}}_1$, which contains only the variable *Line*
- ${\boldsymbol{X}}_2$, which contains the remaining variables *Diet, Order, Block, Concb, RINb, Conca, RINa, Baso, Eosi, Lymp, Mono, Neut, RFI*.

**Fig. 2:**
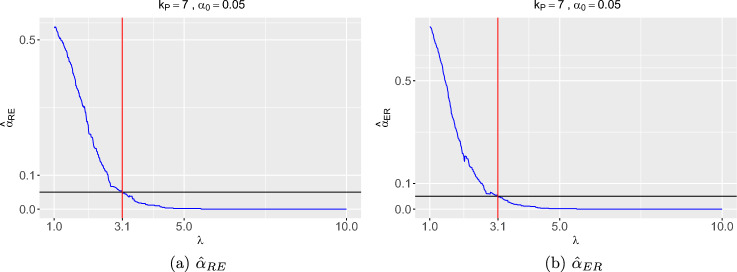
Estimates of false selection rate $\hat{\alpha }_{RE}, \hat{\alpha }_{ER}$ as functions of $\lambda$ when applied to the RFI RNA-seq dataset with $\alpha _0 = 0.05, B = 100$, and $k_P = 7$

We now apply the FSR variable selection method to the RFI RNA-seq dataset. For all scenarios considered below, all methods of generating pseudo-variables yield similar results, except when $\alpha _0 = 0.05$. In this case, the MN mechanism selects one fewer covariate (*RINa*) compared to the other mechanisms. Following the recommendation by Wu et al. (2007) for the case of one response variable and the simulation results in Sect. 4 of this paper, we generate pseudo-variables using the ORX variant method. To estimate $\hat{\alpha }_{\cdot }(\lambda )$, we use $B = 100$ sets of $k_P = 7$ pseudo-variables as $k_P = 7$ gives the best results as shown by our simulation study in Sect. 4. In our analysis, we also consider different FSR thresholds $\alpha _0 \in \{0.01, 0.05, 0.1, 0.2\}$. Figure 2 shows the estimates of $\hat{\alpha }_{RE}(\lambda ), \hat{\alpha }_{ER}(\lambda )$ as functions of $\lambda$. We summarize the removed covariates in Tables 1 and  2. Table 1 presents the covariates removed at each step of the backward selection procedure. For example, *RINb* was the first covariate to be removed from the full model with $r({\textit{RINb}}) = 0.26$, followed by *Eosi*, *Order*, *Conca*, *Diet*, *RFI*, *Lymp*, *Baso*, *RINa*, *Block*, *Neut*, *Concb*, and *Mono* in subsequent iterations.

Table 2 reports the estimates of the critical value $\hat{\lambda }_*$ and the selected variables for each FSR threshold $\alpha _0$. The results are the same for both FSR formula, $\alpha _{RE}$ or $\alpha _{ER}$, therefore, only the results for $\alpha _{RE}$ are displayed in Table 2. The set of selected covariates varies slightly with each FSR threshold. Specifically, for $\alpha _0 = 0.01, 0.05, 0.1$, and $0.2$, the number of selected covariates is 5, 6, 7, and 8, respectively. Notably, the set of covariates selected when $\alpha _0 = 0.1$ is the same as those selected by the backward selection strategy in Nguyen et al. (2015).

**Table 1:** Covariates removed from the full model and their *r* values at each iteration of the backward selection algorithm applied to the RFI RNA-seq dataset

Iteration	1	2	3	4	5	6	7	8	9	10	11	12	13
Covariate	RINb	Eosi	Order	Conca	Diet	RFI	Lymp	Baso	RINa	Block	Neut	Concb	Mono
r	0.26	0.49	0.62	0.65	0.53	2.07	2.87	3.46	6.3	7.71	7.85	9.42	11.45

**Table 2:** Selected covariates using the FSR backward selection algorithm with four FSR threshold $\alpha _0$ = {0.01, 0.05, 0.1, 0.2}

$\alpha _0$	$\hat{\lambda }_{*}$	Selected covariates
0.01	4.26	RINa, Block, Neut, Concb, Mono
0.05	3.10	Baso, RINa, Block, Neut, Concb, Mono
0.10	2.57	Lymp, Baso, RINa, Block, Neut, Concb, Mono
0.20	1.98	RFI, Lymp, Baso, RINa, Block, Neut, Concb, Mono

**Fig. 3:**
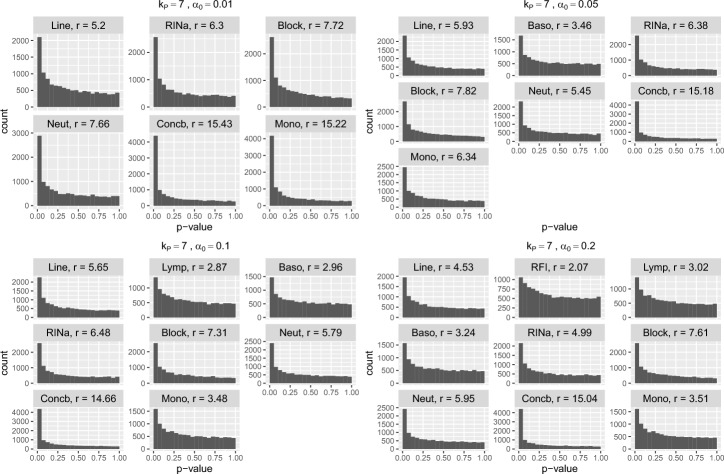
The histograms show the distributions of the *p*-values for the primary variable and the covariates selected by the FSR backward selection algorithm with four options of FSR threshold $\alpha _0 \in \{0.01, 0.05, 0.1, 0.2\}$

Figure 3 displays the histograms of $p$-values of the main factor of interest, *Line*, and the selected covariates when $\alpha _0 \in \{0.01, 0.05, 0.1, 0.2\}$. These histograms have a decreasing shape, indicating that these covariates are highly relevant to the gene expression levels.

As a result, given a false discovery rate of 5%, the model including covariates selected by the FSR method identifies 353, 505, 459, and 50 DE genes for $\alpha _0 = 0.01, 0.05, 0.1,$ and $0.2$, respectively. In contrast, the model including only the *Line* covariate identifies 238 differentially expressed genes, while the model including all covariates identifies none.

## Simulation Study

### Simulation Description

#### Aims

Our simulation study has two main goals. Firstly, we aim to assess the FSR method’s ability to select the most relevant covariates. Secondly, we want to evaluate the FSR-selected model’s ability to identify differentially expressed (DE) genes with respect to the main factor of interest *Line* while controlling false discovery rate (FDR). Additionally, we also investigate the impact of different numbers of pseudo-variables $k_P$ on the performance of the FSR’s method when selecting the most relevant covariates.

#### Data-Generating Mechanisms

To achieve these goals, we require simulated datasets to contain a set of truly relevant covariates and include both EE and DE genes for the primary variables. Due to the complexity of the RNA-seq experiments with multiple available covariates, we will use the RFI RNA-seq dataset for this purpose.

**Table 3:** Six simulation scenarios corresponding to six sets of truly relevant covariates

Number of relevant covariates $k_R$	Relevant covariates
0	
1	Mono
2	Concb, Mono
6	Baso, RINa, Block, Neut, Concb, Mono
7	Lymp, Baso, RINa, Block, Neut, Concb, Mono
8	RFI, Lymp, Baso, RINa, Block, Neut, Concb, Mono

To evaluate a method’s ability to select relevant covariates, we assess its potential to control FSR. We consider the nominal FSR threshold $\alpha _0 = 0.05$ and six sets of $k_R$ relevant covariates, as shown in Table 3. These covariates are chosen based on their levels of relevance when applying the proposed backward selection procedure to the RFI RNA-seq dataset. The first three cases represent scenarios where there are few relevant covariates ($k_R =$ 0, 1, or 2 out of the total 13 covariates), while the last three cases represent scenarios where the relevant covariates ($k_R =$ 6, 7, or 8 out of the total 13 covariates) is large.

The last case with $k_R=8$ relevant covariates is an example in which the relevant covariate *RFI* is strongly correlated with the primary variable *Line* because *RFI* provides a continuous measure of residual feed intake for each of the 31 pigs in the study. The inclusion of the relevant covariate *RFI* makes it difficult to distinguish the effect of *Line* from the effect of *RFI* on expression levels. This difficulty may result in the failure of FDR control (Nguyen et al. 2015).

To evaluate a selected model’s ability to identify DE genes while controlling FDR, we simulated datasets that contain both EE and DE genes with respect to the primary variable with each set of the relevant covariates. To establish realistic parameter values for our simulation, we use parameter estimates from our real dataset – with some regression coefficient estimates close to zero replaced with zero – as true parameter values. Replacing the least significant regression coefficient estimates with zero as true parameter values creates EE genes and addresses the issue that raw estimates from real data are non-zero even when true parameters may be zero. For each simulation scenario, as true parameters to simulate new data, we used the precision weights, the scaled error variances and the partial regression coefficient estimates from the voom-limma fit of the corresponding model to the RFI RNA-seq dataset, except that we set partial regression coefficients on each variable to zero for a subset of genes. More specifically, for each variable $j$ (either relevant covariates or the primary variable *Line*), the ${\hat{G}}_0^{(j)}$ least significant partial regression coefficients were set to zero, where ${\hat{G}}_0^{(j)}$ is the estimated number of the $j$-covariate’s partial regression coefficients equal to zero when the method of Nettleton et al. (2006) is applied to the $j$th variable’s $p$-values from the voom-limma fit of the corresponding model to the RFI RNA-seq dataset. We set all partial regression coefficients to zero for irrelevant covariates. This strategy yielded a parameter vector (consisting of a scaled error variance, precision weights, and partial regression coefficients) for each of 12280 genes. To simulate any particular dataset for a given set of truly relevant covariates, we randomly sampled 2000 gene parameter vectors. The selected parameters and the respective values of the primary variable and the relevant covariates for the 31 pigs were used to simulate a $2000\times 31$ dataset of read counts following the inverse steps of (1) and (2) described in Sect. 2. Random selection of parameters and generation of data was independently repeated 100 times to obtain 100 datasets for each scenario.

In addition to the two aforementioned goals, we examine the impact of the number of pseudo-variables, $k_P$, on the FSR approach. We evaluate four values of $k_P$ – 1, 3, 5, and 7, in which 7 is the maximum number of pseudo-variables that can be added while still preserving at least one degree of freedom for error estimation in the first stage of backward selection.

#### Estimands

For variable selection, our estimands are the false selection rate (FSR) and the number of selected covariates that are truly relevant ($S$). For differential expression analysis, our estimands include the false discovery rate (FDR), the number of correctly declared differentially expressed genes (NTP), and the partial area under ROC curve (PAUC) with false positive rate less than or equal to 0.05.

#### Methods

We explore eight versions of the FSR method that result from combining two FSR estimators, $\alpha _{ER}$ and $\alpha _{RE}$, with four methods of pseudo-variable generation – WN, RX, OWN, and ORX. As noted at the end of Sect. 2, we refer to these methods as WN$\_$RE, WN$\_$ER, RX$\_$RE, RX$\_$ER, OWN$\_$RE, OWN$\_$ER, ORX$\_$RE, and ORX$\_$ER. We also compare our proposed approaches to the backward selection method of Nguyen et al. (2015) that relies on the measure of covariate relevance Nguyen et al. (2015) refer to as $p$.05. In our simulation results, we refer to this method as BS15.

Then, we performed differential expression analysis using covariates obtained from the nine methods above and three other strategies for handling covariates. These three other strategies used models that include all 13 available covariates (Full), only the primary variable *Line* (Line Only), and the true set of covariates used to simulate the count data (Oracle). In total, there are twelve methods for differential expression analysis.

#### Performance Measures

For the analysis strategies, we used the voom-limma pipeline implemented in the R package limma to calculate $p$-values for testing the significance of the partial regression coefficients of each variable in the model. For the primary variable, we then convert these $p$-values to $q$-values, as described in Sect. 2.2. We declared genes as DE if their $q$-values were no larger than 0.05. Otherwise, a gene was declared as EE. For covariates that were subject to selection, we used the $p$-values to calculate the relevance measure $r$, which was subsequently used to calculate the critical value $\lambda _*$ in the FSR backward selection procedure.

We evaluate the empirical estimates of the estimands FSR, S, FDR, NTP, and PAUC along with their standard errors across 100 replications. Regarding variable selection, we determine the average false selection rate (FSR) and the average number of selected covariates (S) over these 100 replications. For the analysis of differential expression, our focus is on the empirical false discovery rate (FDR), the average number of true positives (NTP), and the average partial area under the ROC curve (PAUC) across the same 100 replications. These metrics, respectively, indicate our ability to control error rates, the power to detect DE genes, and the capability to distinguish DE genes from EE genes. Both the nominal levels of false selection rate for variable selection and false discovery rate for differential expression analysis are set at 0.05.

### Simulation Results

**Fig. 4:**
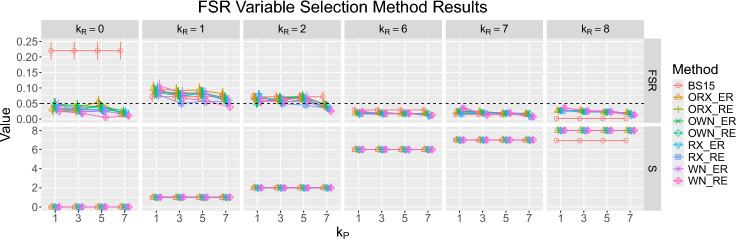
The figure displays the variable selection performance of eight variants of the proposed method and BS15. We consider the nominal false selection rate $\alpha _0= 0.05$ and $k_P \in \{1, 3, 5, 7\}$. There are six simulation scenarios, each with 100 replications, and each replication includes a simulated count data of 2000 genes for 31 samples. The comparison metrics are the empirical false selection rate (FSR), the average number of selected important covariates (S) **and their empirical standard errors (shown as error bars)** over 100 replications

We begin by assessing the nine backward selection methods in terms of FSR control. The results in Fig. 4 show that all eight variants of our proposed method control FSR well when the number of relevant covariates $k_R$ is 6, 7, or 8 or when there are no relevant covariates. When $k_R$ is 1 or 2, using a larger $k_P$ improves the proposed method’s performance. Overall, our method controls FSR better when $k_P$ is large. In all cases, all variants of our method correctly select the true model most of the time, even when $k_R = 8$.

Figure 4 suggests that FSR for BS15 decreases with $k_R$. When $k_R = 8$, FSR of BS15 is 0 because BS15 always selects either 6 or 7 truly relevant covariates in all 100 replications, and *RFI* is never selected. This occurs because the relevant covariate *RFI* is strongly associated with the primary variable *Line* due to genetic selection, as discussed in Sect. 3, and is discouraged in the selection process.

From the results of our simulation study, our proposed method performs similarly in terms of FSR control for different values of $k_P$. Larger $k_P$ tends to show better FSR control when the number of relevant covariates is relatively small. Therefore, we suggest using the largest possible $k_P$ as long as it allows for at least one degree of freedom for estimating error variances in the voom-limma pipeline.

We now turn our attention to evaluating the performance of the twelve differential expression analysis methods described above. As depicted in Fig. 4, even though there is a fairly consistent performance in terms of FSR control with different value of $k_P$, our proposed method performs best when using $k_P = 7$ pseudo-variables. Throughout our analysis of simulated data, we used $k_P = 7$ pseudo-variables for the FSR method.

**Fig. 5:**
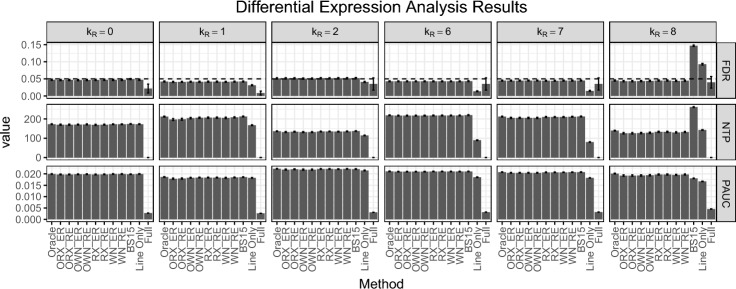
The figure presents the performance of differential expression analysis of the twelve methods. These methods are evaluated under six simulation scenarios, with the nominal false selection rate $\alpha _0 = 0.05$ and $k_P = 7$ pseudo-variables. Each simulation scenario includes 100 replications with a simulated count data of 2000 genes for 31 samples. The comparison metrics include the empirical false discovery rate (FDR), the average number of declared true DE genes (NTP), the average partial area under ROC curve (PAUC) with false positive rate less than 0.05 **and their empirical standard errors (shown as error bars)** over 100 replications

Figure 5 demonstrates that all eight variants of the proposed method effectively control FDR. Moreover, their NTP and PAUC are almost the same as those of the Oracle method. The Line Only method fails to control FDR when $k_R = 8$, while the Full method is able to control FDR conservatively with lower NTP and PAUC. BS15 method performs comparably to our proposed method, except when $k_R = 8$.

### Additional Simulation Study

We also conducted two additional simulation studies, detailed in Sects. 5 and 6 of the accompanying Supplementary Materials. Section 5 explores a simulation study investigating scenarios where the association between the primary variable, *Line*, and the most strongly associated covariate, *RFI*, has been removed. Section 6 presents an analysis and simulation study based on an RNA-seq dataset of zebrafish embryos from a randomized study by Reinwald et al. (2022). The RNA-seq dataset and experimental design are available at https://www.ebi.ac.uk/biostudies/arrayexpress under access number E-MTAB-9852. The findings from these additional studies demonstrate that our proposed methods perform comparably well in terms of variable selection and, consequently, in differential expression analysis.

## Conclusion and Discussion

In this paper, we proposed a novel approach for selecting relevant covariates in RNA-seq differential expression analysis. Our approach is built upon the variable selection method of Wu et al. (2007). While Wu et al. (2007)’s method is designed for a single response variable, our method is applicable to multiple response variables, such as those found in RNA-seq data. Our method successfully identifies relevant covariates, even in the presence of covariates strongly associated with the primary variable. As a result, our method yields highly accurate downstream differential expression analysis and a reliable list of DE genes, as demonstrated by its ability to control FDR and distinguish EE and DE genes. Although the proposed method is used to analyze RNA-seq data, it is also adaptable to other high-dimensional data types with similar structures, generally referred to as omics data.

Our simulation study demonstrates that all mechanisms for generating pseudo-variables perform similarly, in which a larger number of pseudo-variables $k_P$ results a better FSR control. At the cost of higher computational expense, using a larger number of pseudo-variables can result in better FSR control when the number of relevant covariates is relatively small. Therefore, if computational resources are not a constraint, we recommend using the largest possible $k_P$ for our method. However, within the backward selection framework, $k_P$ has to be chosen so that rank of the design matrix consisting of an intercept, the available covariates, and pseudo-variables is smaller than the sample size $n$.

Due to the need to simulate multiple sets of pseudo-variables to estimate $\alpha _{RE}$ and $\alpha _{ER}$, computation time is a concern for our method. In our simulation and analysis, we set the number of sets of pseudo-variables B to 100, which yields stable and consistent results across all variants. To analyze RFI RNA-seq dataset of 12,280 genes and 14 covariates, it takes our method approximately 50 min using a single core on a personal laptop, and about 5 min using 20 cores in parallel.

In this work, we assume that the measured and available covariates are fixed and provide fairly accurate measurements of the respective quantities. However, in many applications, some covariates may be measured imprecisely, leading to measurement error models. When this occurs, variable selection becomes very challenging due to bias in estimation and a loss of power in detecting true relationships between covariates and response variables. Although various authors have attempted to address variable selection in measurement error models, it remains an unsolved issue in many cases (Yi and Buzas 2021). The challenge is even greater when dealing with multiple response variables, such as in RNA-seq data. We hope to explore this issue further and report our findings in a future publication.

Finally, we have so far only focused on variable selection accounting for available and measured covariates to address gene expression level heterogeneity in RNA-seq analysis. In practice, unmeasured factors may also influence expression levels in addition to the available measured covariates, as noted in Leek and Storey (2007). Expanding our variable selection approach to account for hidden unmeasured factors via surrogate variables, as proposed in Leek and Storey (2007), is an important area of research that we plan to explore in future work. This extension will allow us to better handle the complexity of datasets with multiple sources of variation, which is often the case in genomics, and improve the identification of differentially expressed genes.

## Supplementary Information

Below is the link to the electronic supplementary material.

Supplementary file 1 (pdf 505 KB)
