# Supplementary material for: Identifying Relevant Covariates in RNA-seq Analysis by Pseudo-Variable Augmentation
Source: J Agric Biol Environ Stat. 2024 Nov 2;31(2):367–86. doi: 10.1007/s13253-024-00665-3 (PMC13212669; doi:10.1007/s13253-024-00665-3)
Supplement: Supplementary file 1 — (pdf 505 KB) [file 13253_2024_665_MOESM1_ESM.pdf]

Supplementary Materials for "Identifying relevant covariates in RNA-seq analysis by pseudo-variable augmentation"

2024-09-15

1 Description of Variables in the RFI Dataset

$\boldsymbol{x}_{\cdot 1}$ = ***Line*** is the categorical factor of primary scientific interest. Line has two levels, which correspond to the HRFI and LRFI selection lines. Among the 31 pigs in this study, 15 were from the LRFI line and 16 were from the HRFI line.

$\boldsymbol{x}_{\cdot 2}$ = ***RFI*** is a continuous covariate that provides a measure of the residual feed intake for each of the 31 pigs from which blood samples were drawn for RNA-seq analysis. Pigs in the HRFI line tend to have high *RFI* values, while pigs in the LRFI line tend to have low *RFI* values.

$\boldsymbol{x}_{\cdot 3}$ = ***Diet*** is a categorical factor with two levels corresponding to the two diets (high fiber, low energy vs. low fiber, high energy) that were fed to the pigs in this study. Approximately half the pigs within each line were fed each diet. Because RNA-seq analysis was performed on blood samples collected prior to the initiation of the two diets, this factor is not expected to be associated with the transcript abundance levels measured by RNA-seq.

$\boldsymbol{x}_{\cdot 4}$ = ***Baso*** is a continuous covariate that provides a measure of the concentration of basophil cells in the blood sample drawn from each pig.

$\boldsymbol{x}_{\cdot 5}$ = ***Eosi*** is a continuous covariate that provides a measure of the concentration of eosinophil

cells in the blood sample drawn from each pig.

$\boldsymbol{x}_{\cdot 6}$ = ***Lymp*** is a continuous covariate that provides a measure of the concentration of lymphocyte cells in the blood sample drawn from each pig.

$\boldsymbol{x}_{\cdot 7}$ = ***Mono*** is a continuous covariate that provides a measure of the concentration of monocyte cells in the blood sample drawn from each pig.

$\boldsymbol{x}_{\cdot 8}$ = ***Neut*** is a continuous covariate that provides a measure of the concentration of neutrophil cells in the blood sample drawn from each pig.

$\boldsymbol{x}_{\cdot 9}$ = ***Concb*** is a continuous measure of the RNA concentration in each sample before globin depletion (a step that is necessary to focus sequencing efforts on messenger RNA molecules other than highly abundant globin messenger RNA in each blood sample).

$\boldsymbol{x}_{\cdot 10}$ = ***Conca*** is a continuous measure of the RNA concentration in each sample after globin depletion.

$\boldsymbol{x}_{\cdot 11}$ = ***RINb*** is a continuous measure of RNA integrity within each sample before globin depletion.

$\boldsymbol{x}_{\cdot 12}$ = ***RINa*** is a continuous measure of RNA integrity within each sample after globin depletion.

$\boldsymbol{x}_{\cdot 13}$ = ***Block*** is a categorical factor with four levels corresponding to the four blocks used to organize sample collection and processing. Initially, each block involved eight samples, two for each combination of *Line* and *Diet*. One LRFI sample from the first block was removed from the study due to low-quality RNA.

$\boldsymbol{x}_{\cdot 14}$ = ***Order*** is a categorical factor with eight levels indicating the random order samples were processed within each block.

2 Correlations of Variables in the RFI Dataset

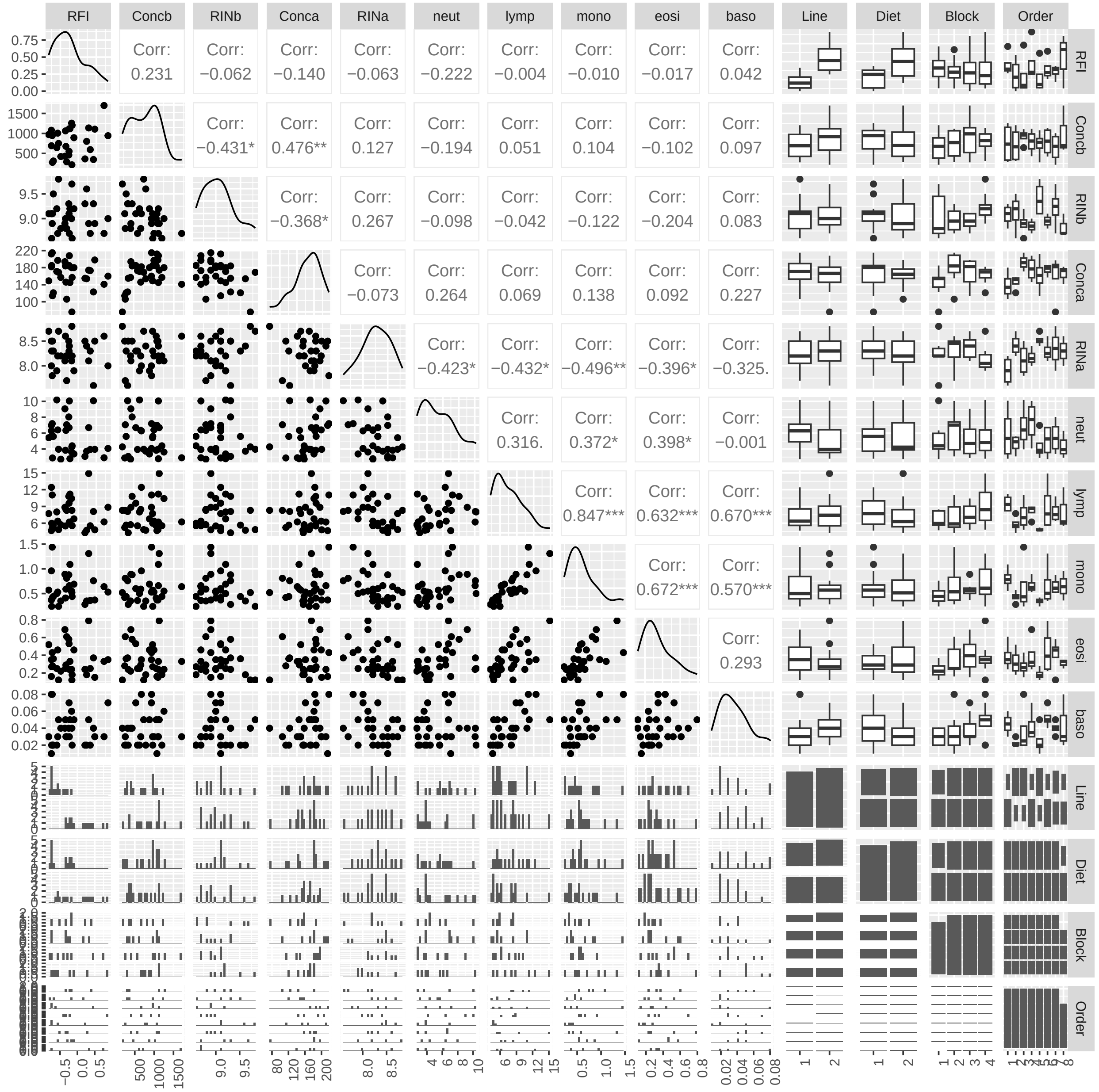

Figure 1: Pairwise correlations of the available variables in RFI dataset.

3 FSR algorithm for estimation of k_I and λ_* using α_{RE}

FSR Procedure Using $\alpha_{RE}(\lambda)$

1. Pick a target false selection rate α_0, e.g., $\alpha_0 = 0.05$.

2. Generate B sets of k_P pseudo-variables. For λ in $\mathcal{A}$, e.g., $\mathcal{A} = \{1, 1.01, 1.02, \cdots, 10\}$, determine the average total number of selected pseudo-variables $\bar{I}^*(\lambda)$ and the average number of selected variables $\bar{S}_P(\lambda)$, then calculate

$$\widehat{\alpha}_{RE,P}(\lambda) = \frac{\bar{I}_P^*(\lambda)}{1 + \bar{S}_P(\lambda)}.$$

3. Obtain an initial cut-off value $c^{(0)}$ from the formula

$$c^{(0)} = \frac{k_P\alpha_0}{k_P\alpha_0 + k_T},$$

   where k_T is the number of real covariates considered for selection. Define $\widehat{\lambda}_*^{(0)}$ as follows

$$\widehat{\lambda}_*^{(0)} = \min\{\lambda : \widehat{\alpha}_{RE,P}(\lambda) \le c^{(0)},\ \lambda \in \mathcal{A}\}.$$

4. Run backward selection on the original set of covariates $\boldsymbol{X}_2$ without pseudo-variables using the relevance-level-to-leave $\widehat{\lambda}_*^{(0)}$. Denote the size of the selected model by $\widehat{k}_R^{(0)}$ and set $\widehat{k}_I^{(0)} = k_T - \widehat{k}_R^{(0)}$.

5. Update the cut-off by

$$c^{(1)} = \frac{k_P\alpha_0}{k_P\alpha_0 + \widehat{k}_I^{(0)}},$$

   and then find

$$\widehat{\lambda}_*^{(1)} = \min\{\lambda : \widehat{\alpha}_{RE,P}(\lambda) \le c^{(1)},\ \lambda \in \mathcal{A}\}.$$

6. Go back to Step 4 and iterate until there is no change in $\widehat{k}_I^{(i)}$. The final $\widehat{\lambda}_*^{(i)}$ is used in a final backward selection on the original set of data.

4 FSR algorithm for estimation of k_I and λ_* using α_{ER}

FSR Procedure Using $\alpha_{ER}(\lambda)$

1. Pick a target false selection rate α_0, e.g., $\alpha_0 = 0.05$.
2. Generate B sets of k_P pseudo-variables. For λ in $\mathcal{A}$, e.g., $\mathcal{A} = \{1, 1.01, 1.02, \cdots, 10\}$, determine the average number of selected pseudo-variables $\bar{I}^*(\lambda)$ and the average total number selected variables $\bar{S}_P(\lambda)$, then calculate
$$\widehat{\alpha}_{ER,P}(\lambda) = \frac{\bar{I}^*_P(\lambda)}{1 + S(\lambda)}.$$
3. Obtain an initial cut-off value $c^{(0)}$ from the formula
$$c^{(0)} = \frac{k_P \alpha_0}{k_T},$$
where k_T is the number of real covariates considered for selection. Define $\widehat{\lambda}_*^{(0)}$ as follows
$$\widehat{\lambda}_*^{(0)} = \min\{\lambda : \widehat{\alpha}_{ER,P}(\lambda) \leq c^{(0)},\ \lambda \in \mathcal{A}\}.$$
4. Run backward selection on the original set of covariates $\boldsymbol{X}_2$ without pseudo-variables using the relevance-level-to-leave $\widehat{\lambda}_*^{(0)}$. Denote the size of the selected model by $\widehat{k}_R^{(0)}$ and set $\widehat{k}_I^{(0)} = k_T - \widehat{k}_R^{(0)}$.
5. Update the cut-off by
$$c^{(1)} = \frac{k_P \alpha_0}{\widehat{k}_I^{(0)}},$$
and then find
$$\widehat{\lambda}_*^{(1)} = \min\{\lambda : \widehat{\alpha}_{ER,P}(\lambda) \leq c^{(1)},\ \lambda \in \mathcal{A}\}.$$
6. Go back to Step 4 and iterate until there is no change in $\widehat{k}_I^{(i)}$. The final $\widehat{\lambda}_*^{(i)}$ is used in a final backward selection on the original set of data.

5 Additional Simulation Study: Orthogonal Covariate RFI

In the RFI RNA-seq dataset, the continuous covariate *RFI* shows a strong correlation with the primary variable *Line*. This correlation arises because LRFI and HRFI lines were created by selecting and mating animals with low and high RFI, respectively, for multiple generations. This resulting partial confounding between the *RFI* covariate and *Line* complicates the differentiation of the direct and indirect effects of *Line* on gene expression levels. To address this issue in an additional simulation study, we follow the same simulation strategy as in the main manuscript, but with a modification: we replace the original *RFI* covariate with a new *RFI* variable that is orthogonal to *Line*. This is achieved by subtracting the average RFI value for each line from the original RFI value of that line. Consequently, after these subtractions, the new RFI values sum to zero within each line, ensuring that the new *RFI* covariate is orthogonal to the *Line* factor.

The results of the additional simulation study are presented in Figures 2 and 3. It's evident that across most cases, all variable selection methods exhibit similar performance regarding false selection rates, consistent with the earlier simulation study. However, when $k_P = 8$, the BS15 method controls FSR well. Consequently, regarding differential expression analysis as shown in Figure 3, all examined methods successfully maintain FDR at the nominal level of 5%. LineOnly and Full methods are more conservative and have smaller power for detecting DE genes.

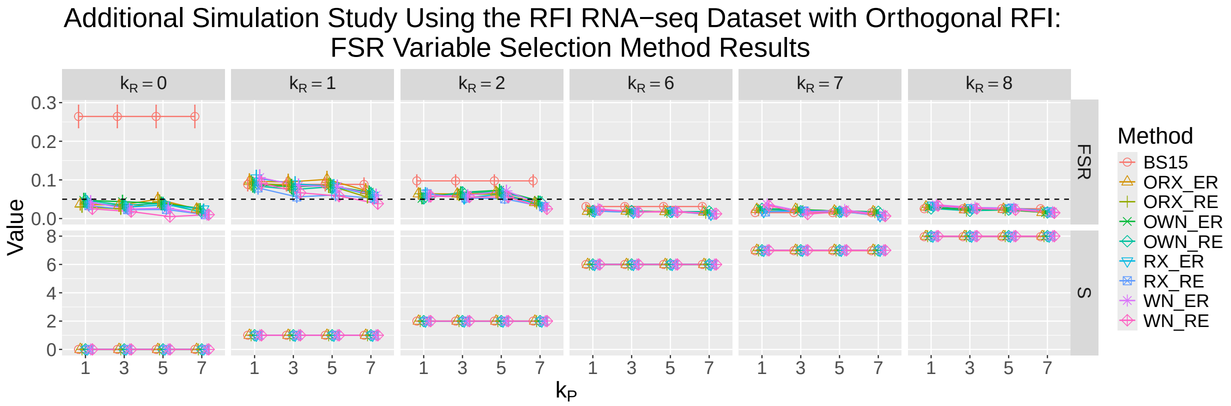

Figure 2: The figure displays the variable selection performance of eight variants of the proposed method and BS15 for the additional simulation study. We consider the nominal false selection rate $\alpha_0 = 0.05$ and $k_P \in \{1, 3, 5, 7\}$. There are six simulation scenarios, each with 100 replications, and each replication includes a simulated count data of 2000 genes for 31 samples. The comparison metrics are the empirical false selection rate (FSR), the average number of selected important covariates (S) and their empirical standard errors (shown as error bars) over 100 replications.

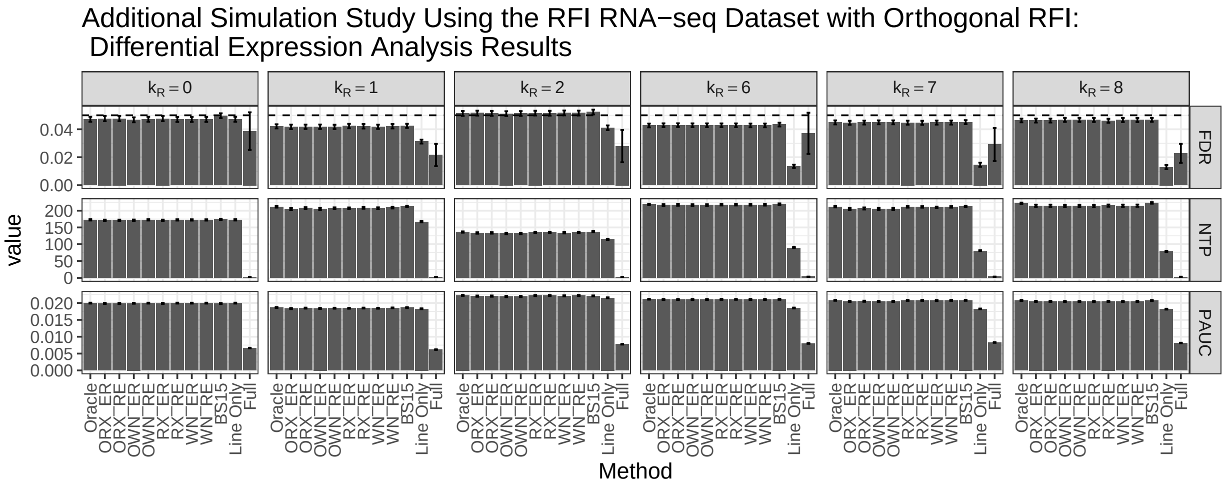

Figure 3: The figure presents the performance of differential expression analysis of the twelve methods for the additional simulation study. These methods are evaluated under six simulation scenarios, with the nominal false selection rate $\alpha_0 = 0.05$ and $k_P = 7$ pseudo-variables. Each simulation scenario includes 100 replications with a simulated count data of 2000 genes for 31 samples. The comparison metrics include the empirical false discovery rate (FDR), the average number of declared true DE genes (NTP), the average partial area under ROC curve (PAUC) with false positive rate less than 0.05 and their empirical standard errors (shown as error bars) over 100 replications.

6 Analysis and Simulation based on a Zebrafish RNA-seq Experiment

In this section, we present an analysis and simulation study based on an RNA-seq dataset of zebrafish embryos from a randomized study by Reinwald et al. (2022).

6.1 Description of the Experiment

The objective of this RNA-seq experiment was to identify ecotoxicogenomic fingerprints in zebrafish embryos exposed to sublethal concentrations of abamectin, an insecticide commonly used in agriculture and horticulture to control various pests. Four exposure concentration levels of abamectin (*Treatment*) were tested: 0 μg/L (normal), 110 μg/L (low), 220 μg/L (mid), 440 μg/L (high). Each treatment level was applied to three biological replicates, resulting in twelve total samples. The biological replicates consisted of eggs from different spawning batches. For each batch, approximately 50 eggs were pre-picked and incubated in 10 mL of the respective test solution. Using stereomicroscopy, 15 fertilized eggs in the early blastula stage (3–4 hours post-fertilization) were selected and transferred to pre-saturated glass dishes containing 8 mL of fresh, aerated test solution. After 96 hours post-fertilization, 10 embryos were randomly selected from each replicate and pooled into a 1.5 mL Eppendorf tube. The tubes were immediately placed on ice to kill the larvae prior to RNA extraction for gene expression profiling. RNA extraction was conducted on February 11 and 18, 2019.

In summary, the final dataset includes 12 RNA-seq samples, each containing 25,390 genes (after filtering out genes with an average expression level below 1), along with associated variables: *Treatment* (the primary factor with 4 levels), *Batch* (a categorical variable with 3 levels), *RIN* (RNA integrity number, a continuous covariate), and *ExtractionDate* (a categorical variable with 2 levels). The RNA-seq dataset and experimental design are available at https://www.ebi.ac.uk/biostudies/arrayexpress under access number E-MTAB-9852.

6.2 Analysis

Figure 4 displays the scatter plot of variables from the Zebrafish RNA-seq dataset. There is no significant association between any of the covariates and *Treatment*. Furthermore, the *ExtractionDate* variable is completely confounded with the *Batch* variable, as all samples from batches T8 and T6 were extracted on February 11, 2019, while the samples from batch T14_3 were extracted on February 18, 2019. Due to this confounding, *ExtractionDate* is excluded from the analysis.

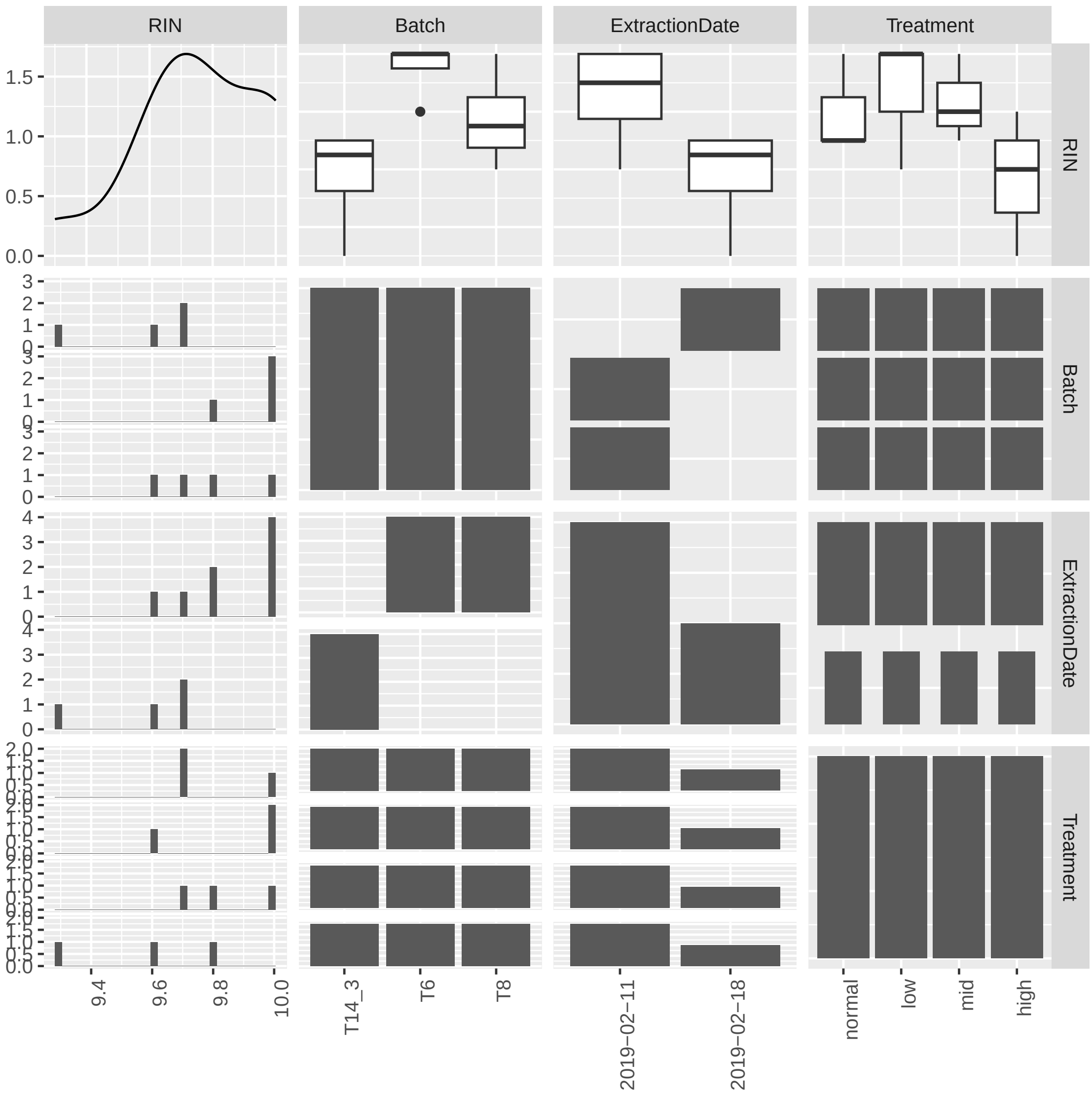

Figure 4: Scatter plot of variables in the Zebrafish RNA-seq dataset.

Table 1: Covariates removed from the full model and their r values at each iteration of the backward selection algorithm applied to the Zebrafish RNA-seq dataset.

Iteration	1	2
Covariate	RIN	Batch
r	1.17	4.16

Table 2: Selected covariates using the FSR backward selection algorithm with FSR threshold $\alpha_0 = 0.05$ for the Zebrafish RNA-seq dataset.

α_0	$\hat{\lambda}_*$	Selected covariates
0.05	1.89	Batch

We now apply our covariate selection procedure to the Zebrafish RNA-seq dataset, where the primary variable is *Treatment* and the covariates are *Batch* and *RIN.* We set the false selection rate nominal level at $\alpha_0 = 0.05$, with $B = 100$ sets of $k_P = 4$ pseudo-variables, the maximum possible for this experiment. Table 1 outlines the covariates removed at each step of the backward selection procedure: *RIN* was removed first, followed by *Batch* in the second iteration. Figure 5 displays the estimates of $\alpha_{RE}(\lambda), \alpha_{ER}(\lambda)$ as functions of λ. Table 2 provides estimate of the critical value $\hat{\lambda}^* = 1.89$ and identifies *Batch* as the selected covariate. The BS15 method also selects *Batch* as the only relevant variable. Along with the primary factor of interest *Treatment, Batch* was the only covariate used in the differential expression analysis by Reinwald et al. (2022), consistent with the finding of our covariate selection method.

Figure 6 shows the histograms of p-values for the primary variable *Treatment* and the selected covariate *Batch.* At a false discovery rate of 5%, the model including covariates selected by the FSR method and BS15 identifies 10,652 differentially expressed (DE) genes among *Treatment* levels. In comparison, the model with only *Treatment* identifies 8,096 DE genes, while the model including all covariates identifies 5,507 DE genes.

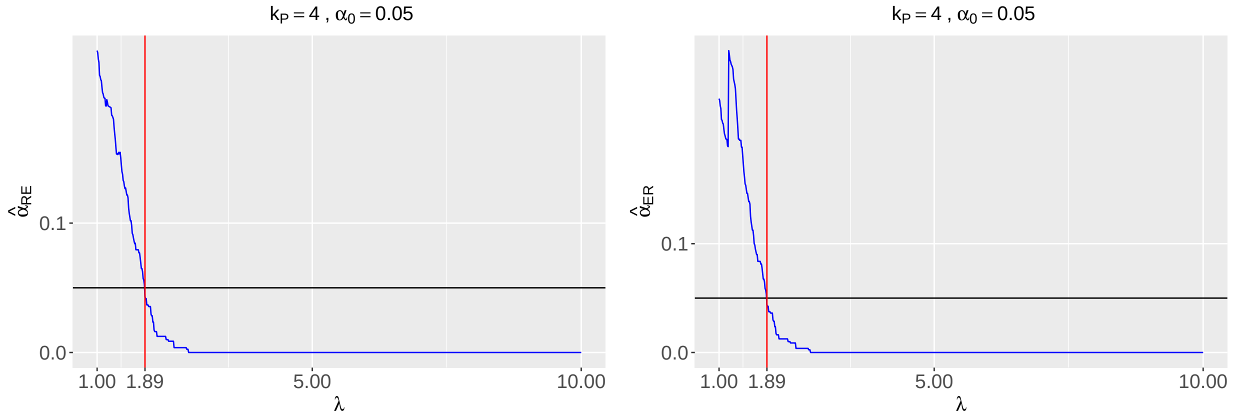

(a) $\hat{\alpha}_{RE}$ (b) $\hat{\alpha}_{ER}$

Figure 5: Estimates of false selection rate $\hat{\alpha}_{RE}, \hat{\alpha}_{ER}$ as functions of λ when applied to the Zebrafish RNA-seq dataset with $\alpha_0 = 0.05, B = 100$, and $k_P = 4$.

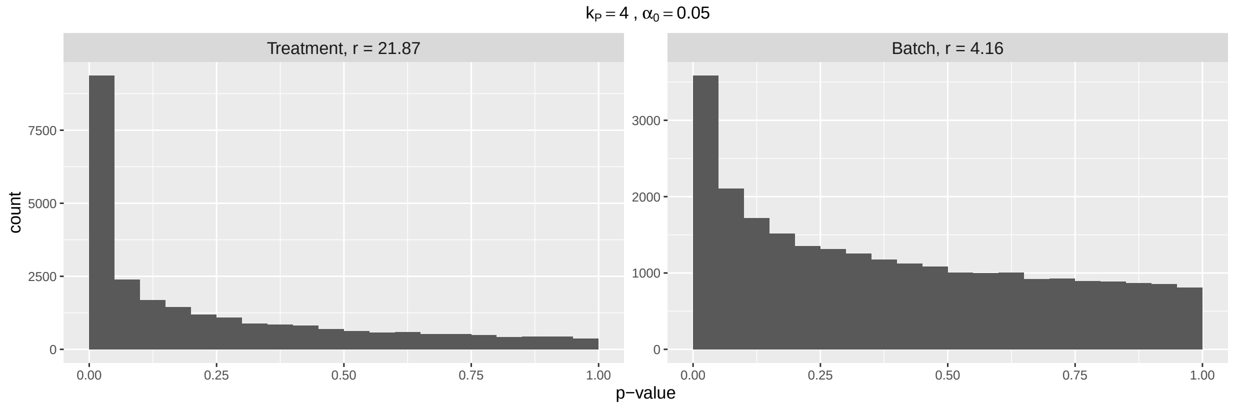

Figure 6: The histograms show the distributions of the p-values for the primary variable *Treatment* and the covariates selected by the FSR backward selection algorithm with FSR threshold $\alpha_0 = 0.05$ using the Zebrafish RNA-seq dataset.

6.3 Simulation Study

Next, we conduct a simulation study based on the Zebrafish RNA-seq dataset. The simulation strategy is identical to that described in the main manuscript, except this time, we use the Zebrafish RNA-seq dataset, where the main factor of interest is *Treatment*, and the available covariates are *Batch* and *RIN*. We consider two sets of truly relevant covariates: $\{\emptyset\}$ ($k_R = 0$) and $\{Batch\}$ ($k_R = 1$) along with four values for the number of pseudo-variables $k_P \in \{1, 2, 3, 4\}$.

The results of this simulation study are shown in Figures 7 and 8. All variable selection methods demonstrate similar performance in terms of false selection rates, with the best performance observed when $k_P = 4$, consistent with the earlier simulation studies. BS15 performs well, as no covariates are strongly correlated with the main factor of interest. Additionally, as illustrated in Figure 8, all methods successfully maintain a false discovery rate (FDR) at the nominal level of 5%. Treatment Only and Full methods are more conservative, showing smaller PAUC and lower power for detecting differentially expressed (DE) genes. These results are consistent with those obtained in Section 5 of this Supplementary Materials.

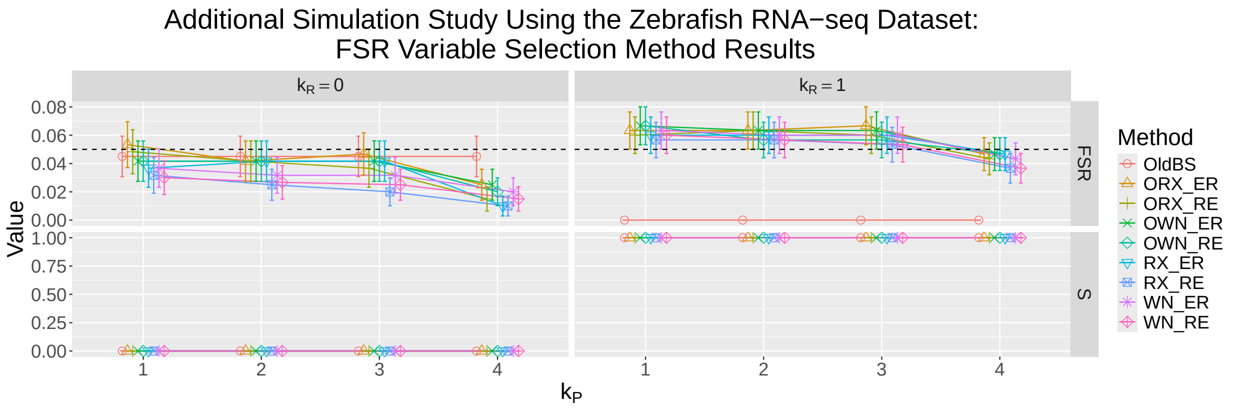

Figure 7: The figure displays the variable selection performance of eight variants of the proposed method and BS15 for the additional simulation study using the Zebrafish RNA-seq dataset. We consider the nominal false selection rate $\alpha_0 = 0.05$ and $k_P \in \{1, 2, 3, 4\}$. There are two simulation scenarios, each with 100 replications, and each replication includes a simulated count data of 2000 genes for 12 samples. The comparison metrics are the empirical false selection rate (FSR), the average number of selected important covariates (S) and their empirical standard errors (shown as error bars) over 100 replications.

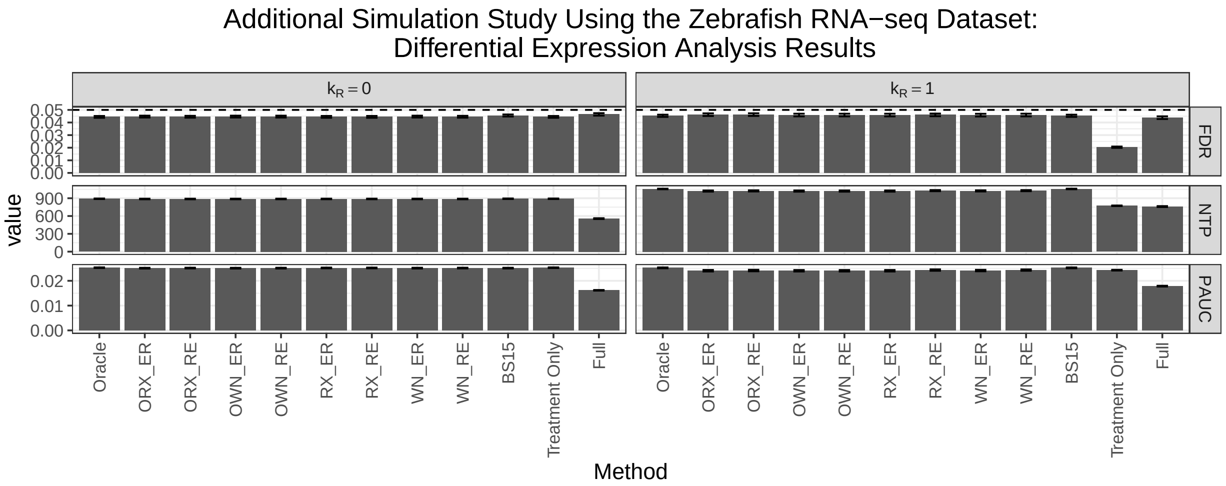

Figure 8: The figure presents the performance of differential expression analysis of the twelve methods for the additional simulation study using the Zebrafish RNA-seq dataset. These methods are evaluated under three simulation scenarios, with the nominal false selection rate $\alpha_0 = 0.05$ and $k_P = 4$ pseudo-variables. Each simulation scenario includes 100 replications with a simulated count data of 2000 genes for 12 samples. The comparison metrics include the empirical false discovery rate (FDR), the average number of declared true DE genes (NTP), the average partial area under ROC curve (PAUC) with false positive rate less than 0.05 and their empirical standard errors (shown as error bars) over 100 replications.

References

Reinwald, Hannes, Julia Alvincz, Gabriela Salinas, Christoph Schäfers, Henner Hollert, and Sebastian Eilebrecht. 2022. "Toxicogenomic Profiling After Sublethal Exposure to Nerve- and Muscle-Targeting Insecticides Reveals Cardiac and Neuronal Developmental Effects in Zebrafish Embryos." *Chemosphere* 291: 132746.
